# Trends in Prevalence of Overweight and Obesity in Danish Infants, Children and Adolescents – Are We Still on a Plateau?

**DOI:** 10.1371/journal.pone.0069860

**Published:** 2013-07-24

**Authors:** Camilla Schmidt Morgen, Benjamin Rokholm, Carina Sjöberg Brixval, Camilla Schou Andersen, Lise Geisler Andersen, Mette Rasmussen, Anne-Marie Nybo Andersen, Pernille Due, Thorkild I. A. Sørensen

**Affiliations:** 1 National Institute of Public Health, University of Southern Denmark, Copenhagen, Denmark; 2 Institute of Preventive Medicine, Bispebjerg and Frederiksberg University Hospital, Copenhagen, the Capital Region, Denmark; 3 Section of Social medicine, Department of Public Health, University of Copenhagen, Denmark; 4 Novo Nordisk Foundation Center for Basic Metabolic Research, Faculty of Health and Medical Sciences, University of Copenhagen, Denmark; The University of Queensland, Australia

## Abstract

**Background:**

After the worldwide steep increase in child and adolescent overweight and obesity during the last decades, there is now evidence of a levelling off in the prevalence in many countries in the Western world.

**Aim:**

To examine whether there still is a plateau in the prevalence of overweight and obesity in Danish children and adolescents, or whether the prevalence is decreasing or rising again.

**Methods:**

The trends in the prevalence rates were based on three data sets providing comparable repeated estimates: 1) the Danish Health Visitors Child Health Database (DHVCHD) with measurements on infant and childhood height and weight from 2002 to 2011 (n up to 39,984), 2) the Danish National Birth Cohort (DNBC) with maternal reports of measured infant and childhood height and weight from 1998 to 2010 (n up to 56,826) and 3) the Danish part of the Health Behaviour in School-aged Children survey (HBSC) with self-reported information on adolescent height and weight from the years 2002 to 2010 (n = 16,557). Overweight and obesity were categorized according to WHO growth standards. Trends were assessed by repeated point estimates and linear regression analyses providing regression coefficients for changes in per cent per year with 95% confidence intervals (CI).

**Results:**

The prevalence rates of overweight and obesity for infants, children and adolescents showed a mixed pattern of decline, stability and increase (ranging from -1.10 through 0.29 per cent per year with CI’s from -3.10 through 2.37). Overall, there were no consistent statistically significant trends upwards or downwards, although some significant downward trends in childhood and adolescence were observed.

**Conclusion:**

This study, based on data from 1998 through 2011, showed that the prevalence rates of overweight and obesity among Danish infants, children and adolescents were largely still on a plateau with tendencies for a decline among children and adolescents.

## Introduction

Since the 1970s, very high and increasing prevalence rates of overweight and obesity among children, adolescents and adults have been reported worldwide in developed countries [1,2] including Denmark [3,4]. Childhood overweight and obesity rates continue to rise in some countries like Mexico, India, China, Canada and Vietnam [5,6], but within the last decades the prevalence has plateaued in several European countries, in the US and in Australia [5,7,8]. Some studies have even reported a decrease in the prevalence [5,9,10]. In Denmark, the picture has been similar with an increase followed by a levelling off from year 2000 and onwards in the prevalence of childhood overweight and obesity [5,11,12].

Overweight and obesity in childhood and adolescence have both short- and long-term adverse physical, psychosocial and socioeconomic consequences [13–17]. The prevalence of overweight is above 20% in many countries, as high as up to 25% in Denmark [12], and up to 37% in the US [5,8], posing a troubling threat to future public health. However, the changes in prevalence have shown a so far unexplained irregular pattern with alternating rises and stable periods [5], which calls for a continued monitoring of the prevalence. Extension of the past evolution of the prevalence may help identifying the determinants of the prevalence.

We aimed to examine the trends in prevalence of infant, childhood and adolescent overweight and obesity in a Danish setting using the most recent available data. 

### Populations and Methods

#### Ethics statement

All participants gave informed consent in the DNBC and in the HBSC. Data were analyzed anonymously in the DHVCHD. The storage and linking of the data was approved by the Danish Data Protection Agency and The Danish National Committee on Biomedical Research Ethics approved the collection of data.

In Denmark there are no National Health Examination Surveys. Therefore the trends and prevalence rates were therefore based on the following three most recent and relevant data sets providing repeated estimates based on presumed comparable selection processes and measurements techniques over time; 1) The Danish Health Visitors’ Child Health Database (DHVCHD) with information on infant and childhood height and weight from 2002 to 2011, 2) The Danish National Birth Cohort (DNBC) with information on infant and childhood height and weight from 1998 to 2010 and 3) the Danish part of the cross-national Health Behaviour in School-aged Children survey (HBSC) with information on adolescent height and weight from the years 2002, 2006 and 2010.

#### Population – The Danish Health Visitors’ Child Health Database (DHVCHD)

The DHVCHD includes data from the Capital Region of Denmark from health visitors’ records on infants and children. Data was obtained both in the child’s first year of life and at school entry. Data was obtained from children from five municipalities surrounding Copenhagen. The children were born between the years 2002 and 2010, and we have data from school entry examinations from the years 2008 to 2011. From birth, Danish infants receive health care visits provided for free by health visitors (specially trained nurses) in the home. At school entry, school nurses examine the children in the school [18]. Less than 5 per 1000 children do not receive these health examinations. The health visitors measured weight and length. The 0-1 year-old children were measured lying on a table and weighed wearing light clothes.

We included children with measurements between age 4-6 months (mean 4.4 months (SD 0.7)) and age 8-10 months (mean 8.3 months (SD 0.5)). This gave us 39,984 4-6-month-old and 5,565 8-10 month-old infants for analyses. The school children were measured by the school nurse without shoes and were weighed wearing light clothes. We included 8,255 children aged 5-7 years (mean 6.0 years (SD 0.5)). Approximately 50 per cent of the children contributed with more than one measurement.

#### The Danish National Birth Cohort (DNBC)

During the years 1996 to 2002, a total of 92,274 women with a total of 100,418 pregnancies were enrolled into the DNBC from all over Denmark. The women were interviewed by telephone twice during their pregnancy (in gestational week 16 and 30), and twice after their pregnancy (6 months and 18 months post-partum). About two-thirds of the women who were invited to participate joined the cohort, which encompasses 30% of all Danish pregnant women during this period. Groups with low socioeconomic resources in terms of education, occupation, income and civil status are underrepresented in the DNBC [19]. More details about the DNBC cohort are presented elsewhere [20,21].

All interviews and questionnaires are available in Danish and English at the homepage: www.DNBC.dk. At the interview 18 months after birth, the mothers were asked to report the length and weight of their child at 5 and 12 months of age, which had been measured by their general practitioner (GP) or by a health visitor as part of the routine health care programme. These measurements were recorded in the “Child’s Book” kept by the parents. The age-span of the weight and length measurements was wide, and we included only children within the age-span of 3–8 months (mean 5.3 (SD 0.5)) and 10–15 months (mean 12.6 (SD 0.6)) of age, respectively. This provided data on 56,826 infants at age 3-8-months and 53,260 children at age 10-15-months for analyses. A 7-year follow-up study of the children was conducted from 2005 to 2010. The parents were asked to report their childrens’ latest measured height and weight. A total of 33 per cent of these measurements were made by a GP or by a health visitor and the rest were made by the parents. The reported data from the 7-year follow-up has been compared with measurements carried out at school entry in a sub-sample of 1,122 children, and the conclusion was that the data were sufficiently accurate [22]. Responses from the follow-up were available for analyses on 53,838 5-8-year-old children (mean 7.05 (SD 0.3)).

#### The Health Behaviour in School-aged Children study (HBSC)

The HBSC is a standardized, international WHO collaborative survey with repeated cross-sectional data collections among 11-, 13- and 15-year-old students in representative samples of schools in the participating countries [23]. Cluster sampling is applied with school being the sampling unit. A nationally representative sample of schools is randomly selected among a complete list of public and private schools. From each selected school, students at the relevant grades are invited for participation. For this study we used data from the Danish contribution to the survey from collections conducted in the years 2002, 2006 and 2010. The response rates ranged from 86.3 to 89.3 per cent. The response rates were calculated as the sum of the correct completed questionnaires among all students enrolled in the participating classes together.

Information on weight and height status was collected from anonymous questionnaires by the items: *How much do you weigh without clothes?*” and “How tall are you without shoes?”

For this study we included adolescents from two age spans; 11–13 years (mean 12.5 (SD 0.9)) and 14–16 years (mean 15.1 (SD 0.7)). This gave a total of 16,557 children and adolescents for analysis.

#### Definition of overweight and obesity

We categorized the childrens’ weight status using the World Health Organization (WHO) Child Growth Standards from 2006 [24] as reference for infant weight and the WHO 2007 growth reference [25,26] for children and adolescents.

According to the WHO Child Growth references, infants are classified as overweight if their age- and sex-specific Body Mass Index (BMI) for age value is greater than 2 standard deviation scores (SDS) above the mean. Infants were classified as obese if their age- and sex-specific BMI is greater than 3 SDSs above the mean. Children and adolescents are classified as overweight if their age- and sex-specific BMI for age value is greater than 1 SDS above the mean and obese if their age- and sex-specific BMI for age value is greater than 2 SDSs above the mean [24,25]. The prevalence rates for overweight include both overweight and obesity.

For comparative reasons we provided figures on child and adolescent overweight and obesity based on IOTF cut points [27] (Figures S1 and S2).

#### Statistical analyses

Age, gender and source specific point estimates of the prevalence were calculated with 95% confidence intervals of the estimates. Overall trends and their linearity were assessed graphically. The PROC REG procedure in the SAS statistical software (ver. 9.2) was used to estimate and test for linear trends across time for all groups defined by data source, age and gender. The procedure fits least-squares estimates to linear regression models with the prevalence rates in per cent as a linear function of measurement year, and thereby providing regression coefficients that are changes in prevalence in per cent per year. To adjust for differences in sample size between observations, the prevalence rates were weighted by the inverse of the standard error in the statistical model, which implies that larger samples are given more weight. A p-value below 0.05 was considered statistically significant

## Results

### Trends in infants’ overweight and obesity

**Figure 1:**
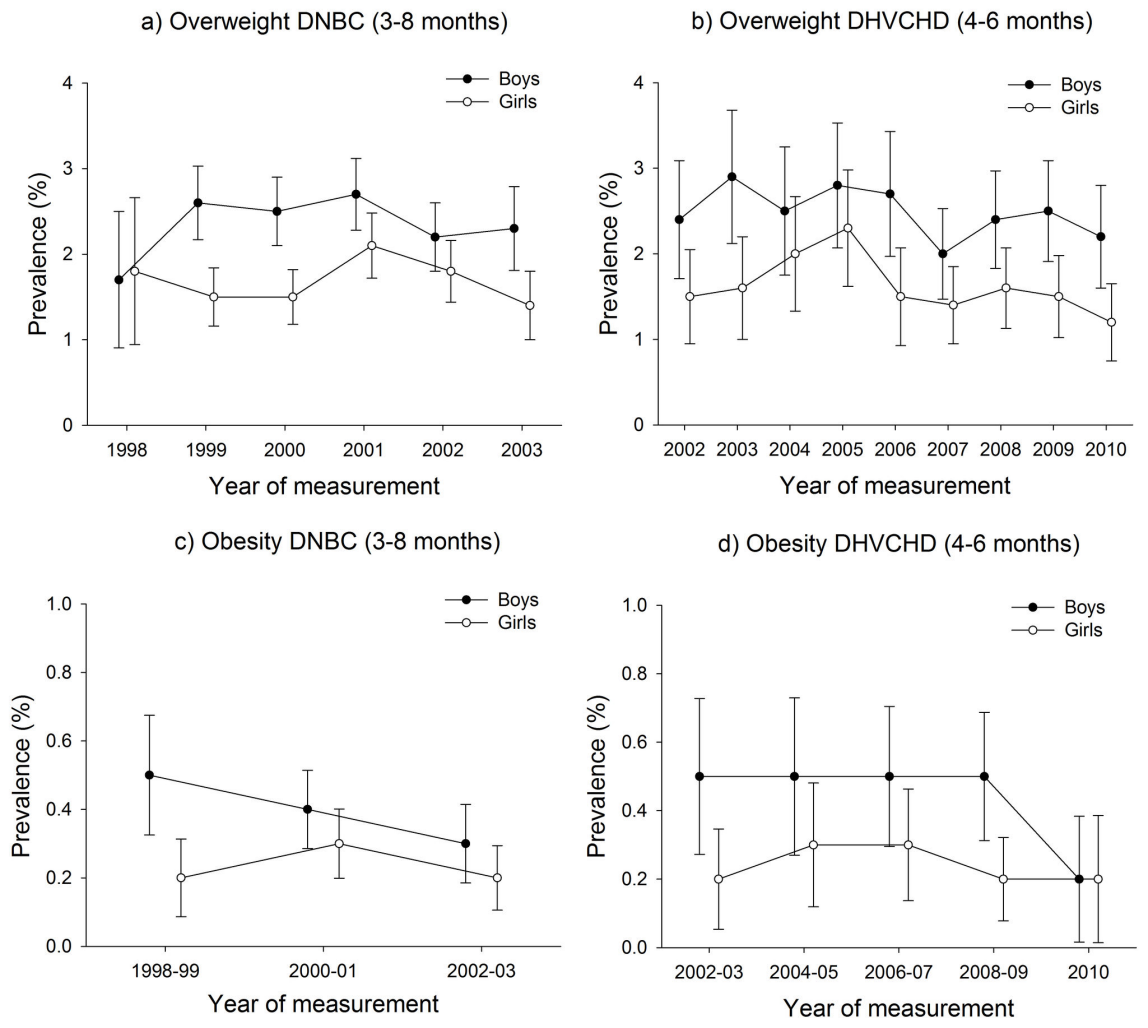
Prevalence of infant overweight and obesity in the DNBC (3-8 months) and in the DHVCHD (4-6 months).

**Figure 2:**
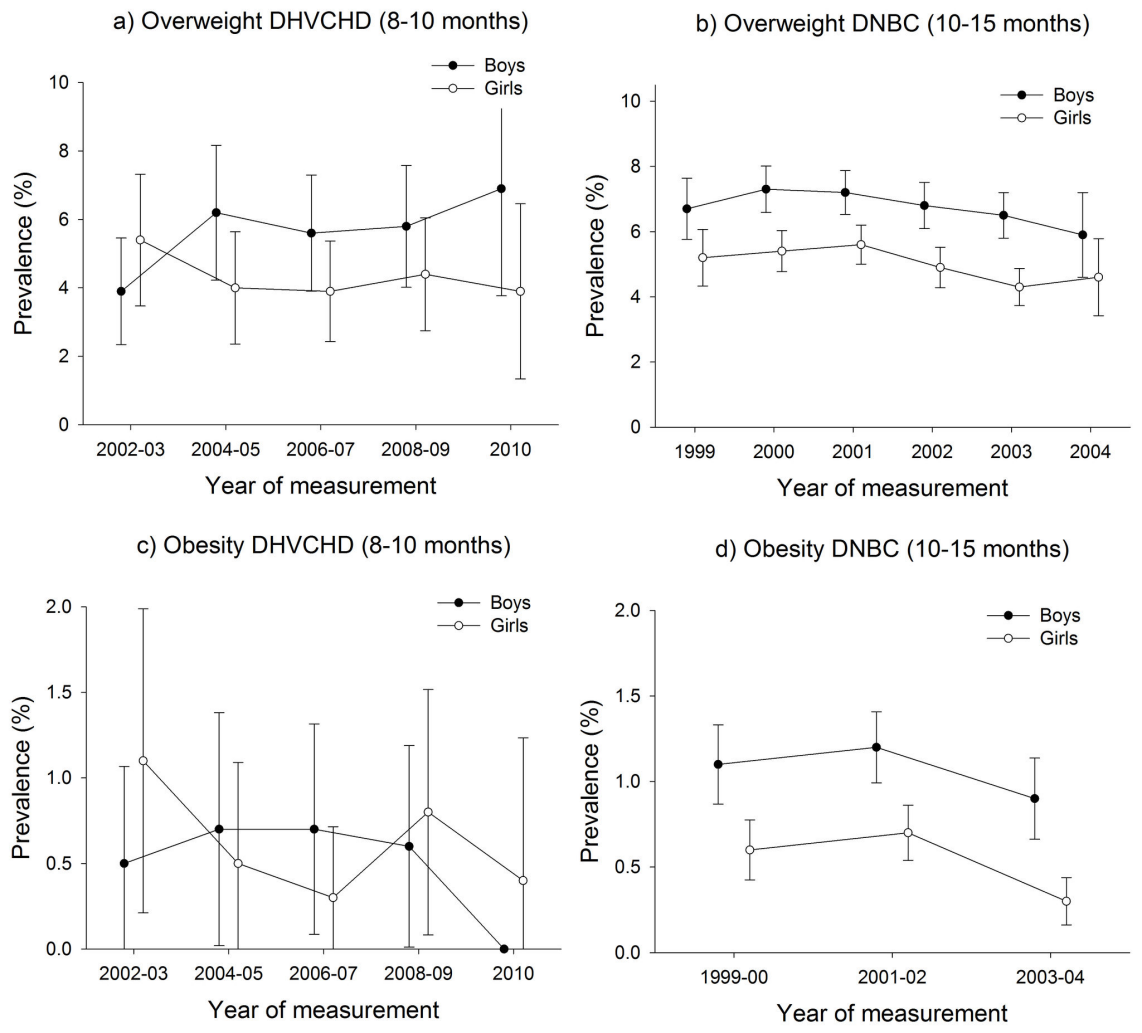
Prevalence of infant overweight and obesity in the DHVCHD (8-10 months) and in the DNBC (10-15 months).

Among all infants (aged 3-15 months) in the study, the prevalence of overweight varied between 1.2 and 7.3%, and the prevalence of obesity varied between 0.0 and 1.2% in the years 1998 to 2010 (Figures 1 and 2).

The overall impression of infant overweight was a slight increase among the 3-8 and 8-10 month-old boys and a slight decrease among the 3-8, 8-10 month-old girls and among the 4-6 and 10-15 month-old boys and girls (Figures 1 and 2). For infant obesity, the figures revealed a tendency towards stability or a decline in all studies and age groups (Figures 1 and 2). However, since all tests for linear trend, except for obesity among 3-8 month-old boys in the DNBC study, were insignificant (β-values in per cent per year between -0.23 (95% CI: -0.50; 0.04) and 0.29 (95% CI: -0.44; 0.19) we concluded that there were no statistical indications of upward or downward trends in the prevalence of infant overweight and obesity during the period of measurement (1998 to 2010) (see table 1).

**Table 1:** Test for linear trends for all groups defined by data source, age, outcome and gender.

	***Datasource***	***Timeofmeasure-ment***	***Agegroup***	***Outcome***	***n***	***Gender***	***β-values*** ^*^	***lower95% CI***	***Upper95% CI***	***p-values*** ^**^
Figure 1.a	DNBC^***^	1998-2003	3-8 months	Overweight	28,933	Boys	0.02	-0.24	0.27	0.87
					27,863	Girls	0.00	-0.22	0.22	0.98
Figure 1.b	DHVCHD^****^	2002-2010	4-6 months	Overweight	20,393	Boys	-0.05	-0,13	0.03	0.21
					19,591	Girls	-0.05	-0,14	0.04	0.22
Figure 1.c	DNBC	1998-2003	3-8 months	Obesity	28,933	Boys	-0.05	-0.07	-0.03	0.02
					27,863	Girls	0.00	-0.37	0.37	0.97
Figure 1.d	DHVCHD	2002-2010	4-6 months	Obesity	20,393	Boys	-0.03	-0.09	0.03	0.18
					19,591	Girls	0.00	-0.03	0.03	0.68
Figure 2.a	DHVCHD	2002-2010	8-10 months	Overweight	2,902	Boys	0.29	-0.13	0.71	0.11
					2,663	Girls	-0.13	-0.44	0.19	0.29
Figure 2.b	DNBC	1999-2004	10-15 months	Overweight	27,029	Boys	-0.19	-0.46	0.08	0.13
					26,204	Girls	-0.23	-0.50	0.04	0.08
Figure 2.c	DHVCHD	2002-2010	8-10 months	Obesity	2,902	Boys	0.02	-0.08	0.12	0.56
					2,663	Girls	-0.05	-0.23	0.14	0.48
Figure 2.d	DNBC	1999-2004	10-15 months	Obesity	27,029	Boys	-0.05	-0.81	0.71	0.56
					26,204	Girls	-0.08	-0.99	0.83	0.46
Figure 3.a	DHVCHD	2008-2011	5-7 years	Overweight	4,225	Boys	-1.10	-3.11	0.92	0.14
					4,030	Girls	-0.32	-1.51	0.86	0.36
Figure 3.b	DNBC	2005-2010	5-8 years	Overweight	24,925	Boys	-0.42	-0.93	0.09	0.09
					26,652	Girls	-0.69	-0.97	-0.42	0.00
Figure 3.c	DHVCHD	2008-2011	5-7 years	Obesity	4,225	Boys	-0.38	-0.88	0.13	0.09
					4,030	Girls	-0.51	-1.97	0.95	0.27
Figure 3.d	DNBC	2005-2010	5-8 years	Obesity	24,925	Boys	-0.18	-0.29	-0.07	0.01
					26,652	Girls	-0.21	-0.39	-0.04	0.03
Figure 4.a	HBSC^*****^	2002-2010	11-13 years	Overweight	6,027	Boys	-0.15	-1.63	1.33	0.42
					6,570	Girls	-0.23	-0.45	-0.01	0.05
Figure 4.b	HBSC	2002-2010	14-16 years	Overweight	4,315	Boys	-0.19	-2.75	2.37	0.52
					4,110	Girls	-0.33	-0.50	-0.16	0.03
Figure 4.c	HBSC	2002-2010	11-13 years	Obesity	6,027	Boys	-0.13	-0.13	-0.12	0.00
					6,570	Girls	0.05	-0.14	0.23	0.19
Figure 4.d	HBSC	2002-2010	14-16 years	Obesity	4,315	Boys	0.11	-1.01	1.24	0.42
					4,110	Girls	0.06	0.59	0.70	0.47

* β-values in per cent per year

** A p-value < 0.05 is considered statistically significant

*** The Danish National Birth Cohort

**** The Danish Health Visitors Child Health Database

***** The Health Behaviour in School-aged Children study

### Childhood overweight and obesity

**Figure 3:**
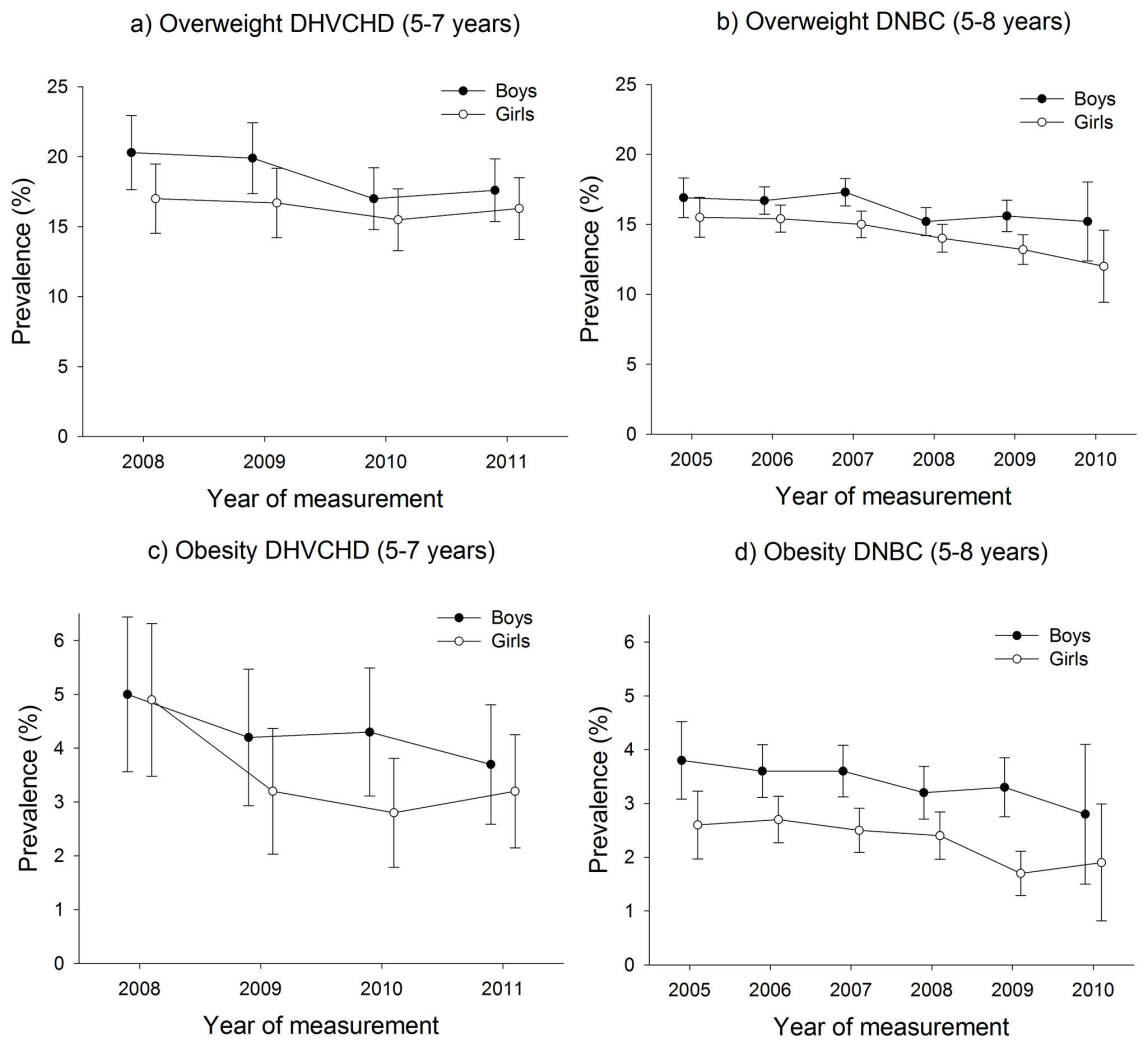
Prevalence of child overweight and obesity in the DHVCHD (5-7 years) and in the DNBC (5-8 years).

Among all children (aged 5-8 years) in the study, the prevalence of overweight varied between 12.0 and 20.4%, and the prevalence of obesity varied between 1.7 and 5.0% (Figure 3).

The overall picture when studying the figures of the prevalence rates of childhood overweight and obesity (Figure 3) was a decrease in both overweight and obesity. However, the tests for trend revealed both statistically significant and insignificant changes. We observed stability for overweight and obesity in the DHCVD study (β-values in per cent per year between -1.10 (95% CI: -3.11; 0.92) and -0.32 (95% CI: -1.51; 0.86), (see Table 1) and we observed a decrease in the DNBC study for overweight among boys (β-value in per cent per year at -0.69, (95% CI: -0.97; -0.42)) and for obesity for boys and girls (β-values in per cent per year at -0.18, and at -0.21 with CI’s from -0.39 through -0.04), (see table 1).

Hence, we concluded that there were tendencies for a decrease in the prevalence of childhood overweight and obesity, which were significant for the DNBC (2005-2010).

### Adolescent overweight and obesity

**Figure 4:**
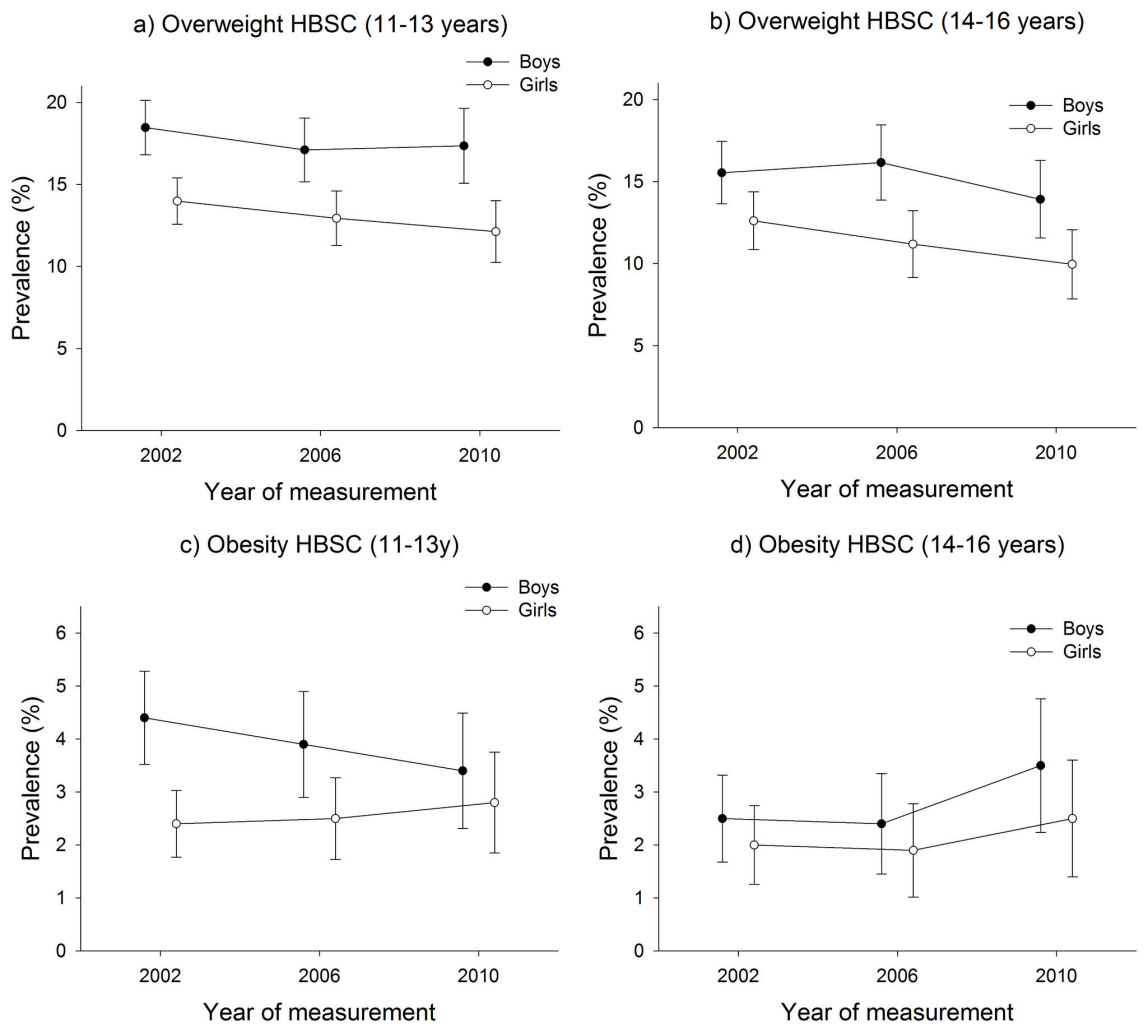
Prevalence of adolescent overweight and obesity in the HBSC.

In the HBSC, the prevalence of overweight among adolescents varied between 9.9 and 18.5%, and the prevalence of obesity varied between 1.9 and 4.4% (Figure 4). The overall impression was a tendency towards a decrease in prevalence rates between 2002 and 2010. The linear regression coefficients for overweight and obesity were statistically insignificant (β-values in per cent per year between -0.33 (95% CI: -0.50; -0.16) and 0.11 (95% CI: -1.01; 1.24) except for overweight among girls in both the 11-13 and 14-16 year age groups and obesity among boys aged 11-13 years (β-values in per cent per year between -0.33 (95% CI: -0.50; -0.16) and -0.13 (95% CI: -0.13; -0.12) (see table 1).

The figures based on International Obesity Task Force (IOTF) cut-offs showed a gender difference when compared to the figures based on the WHO reference but the figures revealed similar trends in the prevalence rates for overweight and obesity among children and adolescents (Figures S1 and S2).

We concluded that for adolescents there was a tendency to a levelling off or even a decline in the prevalence rates for overweight and obesity in the period from 2002 to 2010.

## Discussion

With this study we have described the trends in prevalence of overweight and obesity among Danish infants, children and adolescents with data updated up until 2011. The trends for overweight and obesity among infants, children and adolescents were generally stable, but with some mixed tendencies, some of which were statistically significant. Thus, we found a tendency towards a decline in overweight and obesity in children and adolescents.

Most countries have experienced an increased prevalence of overweight and obesity during the past few decades [1–4,11], but recent data suggest that the prevalence has stabilized or even decreased, as observed in Australia, the US and several European countries [5,8–10]. With the results of this study based on Danish data, we contribute to the growing evidence of the levelling off in the prevalence of overweight and obesity among children and adolescents that now seems to be occurring simultaneously in many parts of the world [5].

### Possible explanations for the observed levelling off

It is yet unclear why we see a levelling off. One reason could be that child and adolescent overweight and obesity has been recognised as a major public health concern, which has led to increased focus on healthy eating and physical activity among the health providers and the general population. However, it remains unclear if this is the reason because of inconsistent evidence for sustainable efficacious intervention and prevention [6,28]. Moreover, in Denmark, after an initial rise of the prevalence among those born during the 1940’es a period of levelling off has been observed, while the typical features of the obesogenic society were emerging [29].

Another reason could be that we have reached a saturation equilibrium such that any child with a predisposition to become overweight have already become overweight. Though since the levelling-off has occurred at very different levels in different countries and since the prevalence rates in Denmark have levelled off at a lower level than in other countries we do not consider this as a possible explanation.

Since the prevalence of overweight and obesity is a combination of the incidence and the duration of the condition, a stable prevalence may not necessarily indicate that these two factors are both stable. In theory, it could mean that the incidence continues to be increasing while the duration of the condition is shortening. A study addressing the question of incidence and persistence revealed that during the early phases of the obesity epidemic, both incidence and duration of overweight and obesity increased. However, a remission rate of obesity in more than 60% of obese children indicated that the condition is not necessarily stable [30]. We do not know the dynamics behind the recent generally stable prevalence in Denmark, and we still need to uncover these in order to understand the trends reported here and in similar studies. The levelling off in the overall picture may hide opposing trends between socioeconomic groups. In this case, one could speculate that the socioeconomically advantaged groups have been able to reduce both the incidence and duration of the condition, while the socioeconomically disadvantaged have not, which may have led to an increasing socioeconomic inequality in infant, child and adolescent overweight [8,31]. Some of the trends observed in the present study may have this background, and it would be very relevant to investigate in future studies.

The stabilisation could be due to a self-selection bias, if greater awareness and possibly greater stigmatisation has led to greater reluctance to participate in surveys, or if there are temporal trends in misclassification of reported height and weight [32]. However we do not consider this to have biased our results since we have based our conclusions on routinely collected measured data also.

The epidemic of obesity has been shown to develop in phases alternating with one generation apart between stable and increasing periods closely linked to birth cohorts [33]. The present period with stable prevalence may have started among those born in the early 1990’es and may, possibly by trans-generational mechanisms, be linked to the previous period with a stable prevalence among those born between the early 1950es through the late 1960es.

### Perspectives for the future

Continuous collection of anthropometric data enables us to more accurately identify possible changes in trends, which may again provide useful insight into what could be the determinants of the changes in the prevalence. Furthermore, studying trends continuously in different settings with differential exposures to the obesogenic environment and differences in norms, health policies and prevention strategies may bring us closer to an understanding of which determinants are behind the development of infant, child and adolescent overweight, and which preventive initiatives that may have been successful on a population level.

### Strengths and limitations

Our study has strength and limitations that need to be considered to qualify the conclusions. The current study is the collection of data from recent years from various sources, and the conclusions were based on large data samples with a minimum of three consecutive comparable sets of height and weight. This enabled us to update previous prevalence papers by examining the most recent trends in Denmark. Objective measures of height and weight were collected from both the DNBC and the DHVCHD.

The data from the HBSC are randomly selected from all over Denmark, and are considered to be representative for schools in Denmark. Data in the DHVCHD study were collected in the municipalities in the capital region of Denmark. Further data from the DNBC are collected from all over Denmark, and all together the data materials available enabled us to cover most regions of Denmark. On the other hand, data from the DHVCHD and from the DNBC cannot be considered representative for the entire Danish population with regard to socioeconomic composition. Data from the DHVCHD is routinely collected and covers almost all children, but the municipalities are not representative for all municipalities in Denmark. Further, mothers participating in the DNBC represent a higher socioeconomic position than the source population [19]. However, since we are looking at trends, the possible underrepresentation of children and families from lower socioeconomic groups would only introduce bias if there were trends in selection processes or in reporting accuracy by socioeconomic groups, which we have no reason to suspect.

One limitation is the parental reports of height and weight for the 7-year-old children in the DNBC and the self-reported data for adolescents in the HBSC [34,35]. Though the DNBC data were validated and found to be sufficiently accurate [22] there is a potential underestimation of the number of children who were overweight [36]. This would only introduce bias in the trends if a possible underreporting has changed during the period, which seems unlikely.

The use of BMI as a measure of weight status has been criticized, especially in children, because BMI may be affected by skeletal structure and muscle mass [37]. However, BMI in this age group is highly correlated with the body fat mass, and BMI is considered appropriate for monitoring and comparing the prevalence on a population level [38,39].

The references, cut-offs and the terminology used to define childhood overweight and obesity vary considerably [40] and there is an ongoing discussion about which reference or standard to use [26]. We chose to categorize overweight and obesity according to the WHO growth references rather than by the IOTF references 40 because the former are available for children aged less than 2 years [39]. In order to compare our prevalence rates with other materials we provided supplementary figures for the children and adolescents based on IOTF cut-offs. These revealed different patterns for boys and girls but the overall trend of a levelling-off in the prevalence rates was similar to the figures based on the WHO reference.

## Conclusion

Overall, the prevalence rates of infant, child and adolescent overweight and obesity, categorized according to WHO growth references, largely remained stable during the study period, though with tendencies towards a decline among children and adolescents.

## Supporting Information

Figure S1 Prevalence of child overweight and obesity (IOTF) in the DHVCHD and in the DNBC. (PDF)

Figure S2 Prevalence of adolescent overweight and obesity (IOTF) in the HBSC. (PDF)
