# Supplementary material for: Trends in Prevalence of Overweight and Obesity in Danish Infants, Children and Adolescents – Are We Still on a Plateau?
Source: PLoS One. 2013 Jul 24;8(7):e69860. doi: 10.1371/journal.pone.0069860 (PMC3722196; doi:10.1371/journal.pone.0069860)

Supplementary figure 1. Prevalence of child overweight and obesity (IOTF) in the DHVCHD, 5-7 years (panel a & c) and in the DNBC, 5-8 years (panel b & d).

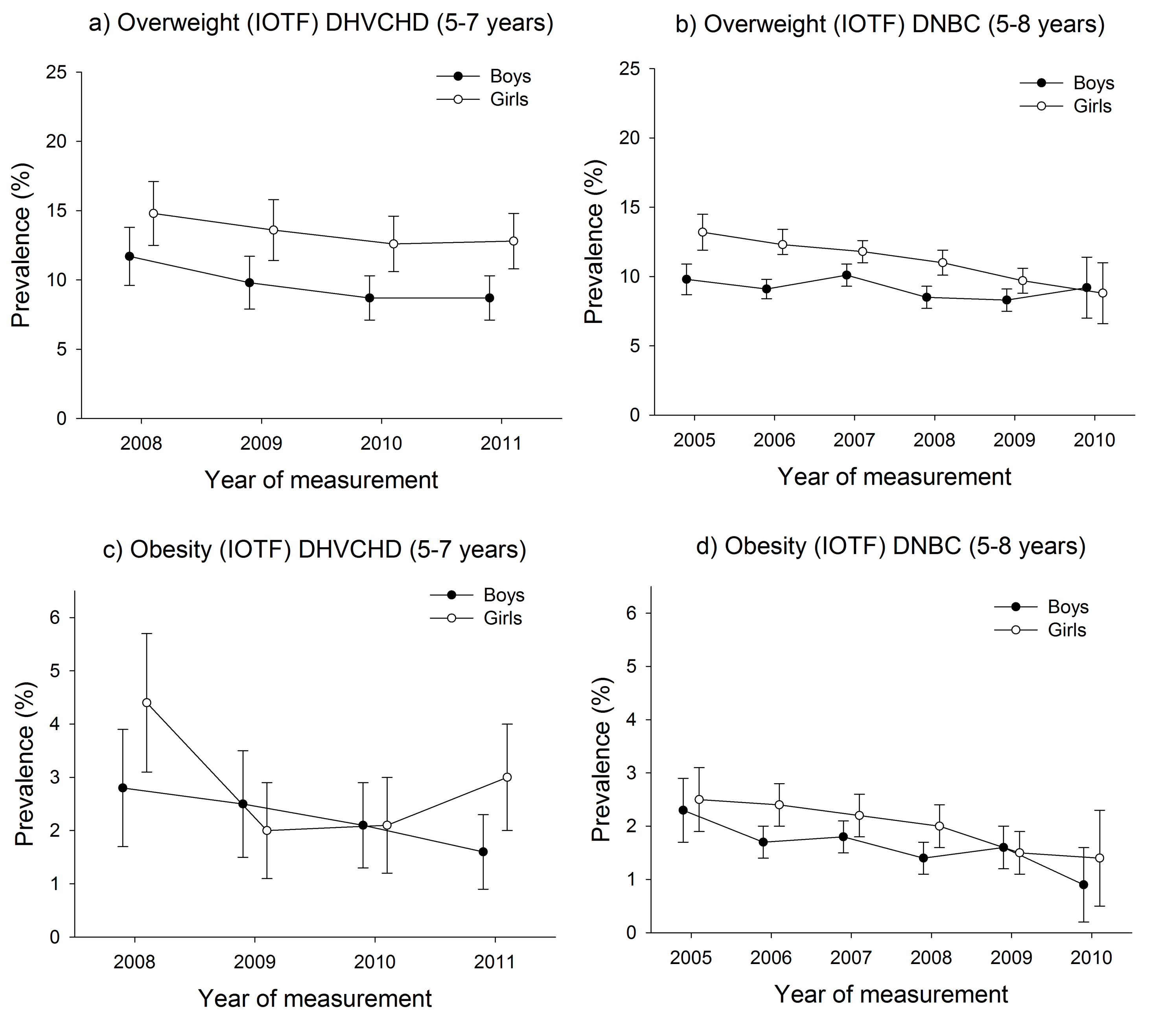

Supplement: Figure S1 — (PDF) [file pone.0069860.s001.pdf]
