# Supplementary material for: Trends in Prevalence of Overweight and Obesity in Danish Infants, Children and Adolescents – Are We Still on a Plateau?
Source: PLoS One. 2013 Jul 24;8(7):e69860. doi: 10.1371/journal.pone.0069860 (PMC3722196; doi:10.1371/journal.pone.0069860)

Supplementary figure 2. Prevalence of adolescent overweight (panel a & b) and obesity (panel c & d) (IOTF) in the HBSC.

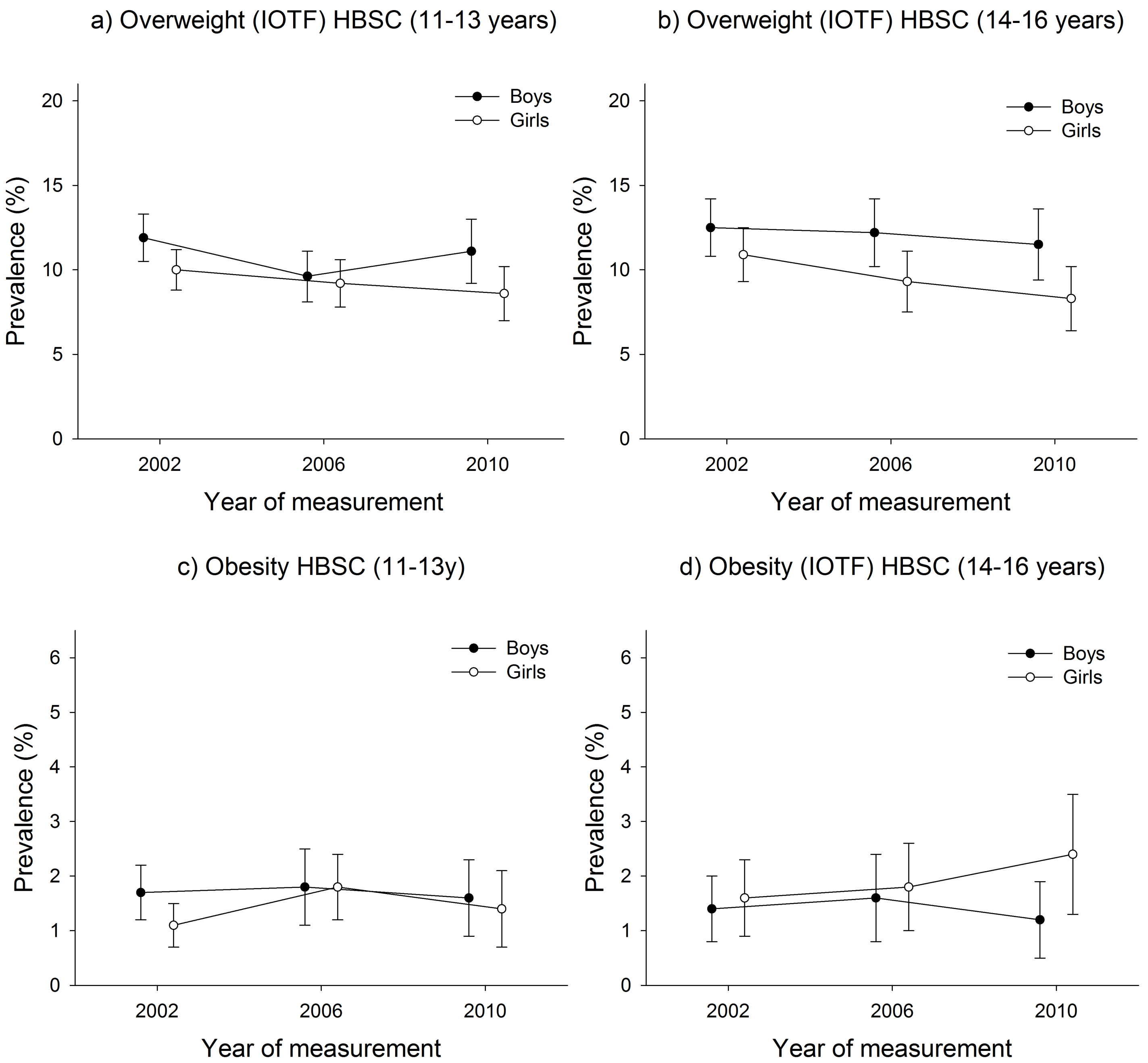

Supplement: Figure S2 — (PDF) [file pone.0069860.s002.pdf]
